# Rat superior colliculus neurons respond to large visual stimuli flashed outside the classical receptive field

**DOI:** 10.1371/journal.pone.0174409

**Published:** 2017-04-05

**Authors:** Juntaute Bytautiene, Gytis Baranauskas

**Affiliations:** Neurophysiology laboratory, Neuroscience Institute, Lithuanian University of Health Sciences, Kaunas, Lithuania; Universidad de Salamanca, SPAIN

## Abstract

Spatial integration of visual stimuli is a crucial step in visual information processing yet it is often unclear where this integration takes place in the visual system. In the superficial layers of the superior colliculus that form an early stage in visual information processing, neurons are known to have relatively small visual receptive fields, suggesting limited spatial integration. Here it is shown that at least for rats this conclusion may be wrong. Extracellular recordings in urethane-anaesthetized young adult rats (1.5–2 months old) showed that large stimuli of over 10° could evoke detectable responses well outside the borders of ‘classical’ receptive fields determined by employing 2° – 3.5° stimuli. The presence of responses to large stimuli well outside these ‘classical’ receptive fields could not be explained neither by partial overlap between the visual stimulus and the receptive field, nor by reflections or light dispersion from the stimulation site. However, very low frequency (<0.1 Hz) residual responses to small stimuli presented outside the receptive field may explain the obtained results if we assume that the frequency of action potentials during a response to a stimulus outside RF is proportional to the stimulus area. Thus, responses to large stimuli outside RF may be predicted by scaling according to the stimulus area of the responses to small stimuli. These data demonstrate that neurons in the superficial layers of the superior colliculus are capable of integrating visual stimuli over much larger area than it can be deduced from the classical receptive field.

## Introduction

To recognize complex images, visual system must integrate responses to visual stimuli well beyond the borders of the classical receptive field (RFs) of a single neuron [1, 2]. The best-known example of such visual stimuli integration is surround suppression when a stimulus outside the excitatory RF suppresses a response induced by a stimulus in the RF center [3–5]. In vision research a receptive field (RF) is defined as ‘the region of visual field over which one can influence the firing of that cell’ [3]. According to this definition, the area that can induce suppression of the responses to a stimulus in the RF center should be considered as part of RF. However, usually only the excitatory part of RF, in which stimuli alone can induce detectable responses, is usually called RF or ‘classical’ RF. This narrow meaning of RF will be used throughout this paper. The influence of stimulation outside RF area on the response properties in RF has been studied quite extensively [6–8]. Although in lateral geniculate nucleus most effects are suppressive [7, 9], data from primary visual cortex indicate that stimuli outside ‘classical’ RF can also enhance responses to a stimulus in RF when co-oriented gratings outside RF are presented [10, 11]. In addition, stimuli outside RF are able to modulate correlation strength between neurons [12]. These and similar data lead to proposal that such background influences can contribute to divisive normalization that requires visual stimuli integration over a large fraction of the visual field [13].

Primary visual cortex does not receive a direct input from retina, its neurons are mainly driven by geniculocortical pathway aided by a large number of feedback pathways from several cortical areas [6, 14]; therefore, the ability of cortical neurons to integrate visual information over large areas is not surprising. In contrast, neurons in the superficial layers of the superior colliculus (SC) receive mostly direct retinal inputs. Although a significant input from primary visual cortex is also present [15], because of the apparent prevalence of retinal inputs in shaping responses of the superficial SC neurons [16–18], one might expect a more limited spatial integration of visual information in these cells. The vast majority of studies that investigated the RF properties of these neurons in many species, including rodents, seem to support this notion [17, 19–22]. These studies indicate that the majority of superficial SC neurons have a simple excitatory RF with an inhibitory surround [19, 20] although they may respond non-linearly to gratings [17]. There is an exception to this simple picture of RF properties in SC neurons. According to one study, in the superficial granular layer I, the most superficial layer in SC, neurons responded to changes in the background illumination and had no well-defined RF [23]. However, the authors of the study claim that most response properties of these neurons to visual stimuli differ from deeper layer SC neurons [23] and we can't extend these findings to other SC neurons. Therefore, to quantitatively evaluate if other neurons in the superficial SC layers can integrate visual stimuli outside the 'classical' RF, responses in the rat SC neurons to small stimuli were compared to the responses to large stimuli inside and outside RF area, which was determined with a small stimulus (<5°). The obtained results show that a two-dimensional Gaussian fit function to the responses to small stimuli fails to predict the amplitude of the responses to large stimuli outside RF indicating that these neurons can integrate over much larger area than it can be deduced from the 'classical' RF.

## Materials and methods

All procedures were carried out in accordance with the European Communities Council Directive of 24 November 1986 regarding the protection of animals used for experimental and other scientific purposes (86/609/EEC) and were approved by the Animal Care and Use Committee of the State Food and Veterinary Service of Lithuania (No. 0239 of 12 September 2012). Male rats weighting 175 to 250 grams, corresponding to the age of 1.5–2.0 months, were anesthetised with aqueous solution of urethane (1.2–1.5 g kg^-1^) delivered intraperitoneally. To relieve the remaining signs of pain-related suffering, a dose of butorphanol (0.4 g kg^-1^) was added intraperitoneally. For the duration of the experiment, the depth of anaesthesia was monitored by testing for the absence of hind limb withdrawal reflex and was maintained by additional doses of butorphanol (i.p.). Under anaesthesia, the body temperature was maintained at 36–38°C with a heating pad. The anesthetized animal was placed in a modified stereotaxic apparatus (World Precision Instruments, Sarasota, FL, USA). The purpose of modifications was to minimize the obstruction of the right eye view; to this end the frame was inverted that allowed to have unobstructed right eye view up to 10 degrees below horizontal plane and a nearly 120 degrees view in the horizontal plane: >90 degrees in the nasal direction to almost 30 degrees in the caudal direction. Eye gel was applied to avoid eye drying. Although in anesthetised rats eye movements are rarely a problem [16], to prevent any eye movements and to maintain lids open, miniature hooks were inserted between the conjunctiva of the inner eyelids and the sclera of the eyes and then attached to the stereotaxic frame with a thread. To dilute eyes, in the majority of experiments atropine solution of 0.5% was applied to eye surface.

For recording, a small craniotomy (approximately 2x2 mm) was made in the parietal bone to expose area above the superior colliculus contralateral to the right eye, identified by vascular landmarks and stereotaxic coordinates of 1.5–3 mm in nasal direction from liambda and 1.0–2.5 mm from the midline [24–26]. Dura mater was left intact. Tetrodes from Thomas Recording (Giessen, Germany) were used to acquire data. Electrodes were typically placed 1.5–2.5 mm rostral to liambda and 0.7–1.9 mm lateral to the midline and then lowered perpendicular to the cortical surface by means of a micro-drive to a depth of >~2000 μm. When the estimated SC border was close, the tetrode was lowered in small steps of 10–30 μm. The presence of the superior colliculus was identified by characteristic spontaneous activity and robust responses to visual stimuli such as movements of small bright spots. At the SC border no separate action potentials but a clear increase in the multi-unit event frequency could be detected. All recordings were performed at the depths of more than 50 μm but less than 350 μm from the superior colliculus border/surface. Lesion tests (n = 3) confirmed that the estimated depth from the SC border was correct and that the recordings were made in the superficial layers of the superior colliculus. For lesions a current of 5–15 μA was passed for 20–40 s through one of the four electrodes of the tetrode.

For extracellular signal acquisition a 4-channel differential amplifier was used (EX4-400, DAGAN, Minneapolis, MN, USA) with a band-pass filters set to 300–10,000 Hz. Data were acquired via a National Instrument card to a PC at 40 kHz sampling frequency and visualized by employing a custom program written in the Labview environment (National Instruments, Austin, TX, USA).

### Visual stimulation

A LED backlit LCD monitor (frame rate 60 Hz, 58 cm by 28 cm) was used for image presentation and was placed 22 cm from the rat right eye slightly below rat’s plain at 45 degrees angle to the rat’s longitudinal axis in the horizontal plain. In the vertical plain the computer monitor was inclined at 30 degrees towards the rat in order to cover a wider range of vertical angles. The full screen subtended 110 horizontally and about 80 degrees vertically. At the center of the screen 1 cm corresponded to ~2.6° of visual angle. Rat eyes are normally emmetropic and can see sufficiently well from 7 cm to infinity [27]. The monitor had 1920 X 1080 image pixels or >30 pixels/cm corresponding to a minimal stimulus size of <0.1°, well below visual acuity of ~0.5° – 1° found in rodents [28]. Images were generated by employing an open source software package PsychoPy controlled by an in house program written in the Labview environment (National Instruments, Austin, TX, USA) [29, 30].

All visual stimuli were bright images, ~30–45 lx at rat’s eye level, presented on a dark grey background (~0.35–0.45 lx). The following *visual stimulation protocols* were used. To determine the size and location of RF, 2.1° – 4.2° wide bright round spots were flashed for 600 ms followed by a 900 ms pause, i.e. stimuli were presented at 1.5 s interval. These spots were flashed on a 18x11 grid in a quasi-random fashion. The grid spacing corresponded to 7.5° and covered most of the monitor. To evaluate an increase in RF area, large stimuli of 15° were presented for 0.5 s or 1 s on a 5x5 grid and then were followed by a 6–9 s pause. To evaluate possible influence of response magnitude adaptation/habituation that could occur with more frequent stimulus presentation [31, 32], for a number of units (first 6 units in S1 Table) small stimuli were presented at 3 s intervals on a smaller 10 by 10 grid with 7.5 degree spacing while large stimuli were presented at shorter intervals, down to 2 s. No obvious changes in RF area were noted with this type of stimulation. In addition, in all remaining experiments, to evaluate the sensitivity to different stimulus size, bright round spots of randomly selected diameter were flashed for 1 s at 7–10 s intervals. No spike rate increase to small stimulus was detected when compared with 1.5 s interval stimulation used in RF area determination. Thus, at least under our experimental conditions in the superficial SC cells, no adaptation to small size stimuli could be detected. We cannot exclude that the response magnitude to large size stimuli was not reduced during frequent stimulation. However, for large size stimulus slow scanning was always employed, in part because of a slow component present in responses to large stimuli (see Results for an example of such a slow response) that complicated the analysis of data during frequent stimulus presentations. On the other hand it was not feasible to have such a long interval for small stimulus because of a denser grid that resulted in several fold higher number of test points and much longer test times. Since stable recordings rarely exceeded few hours, the use of longer intervals on 198 points of the 11 by 18 grid could result into insufficient number of tests in each location. To test contrast sensitivity, stimuli of 7.5° or 15°, presented in RF, were flashed for 1 s each 7 s. Stimulus contrast was changed by maintaining the same background color but changing the spot color. The difference in RGB units was considered as a contrast. Because of RGB signal nature, the minimal contrast was equal to 1/250 of the maximal contrast. For multiple scanning outside RF by small stimulus that was required to estimate the residual responses outside RF, a 2° - 4° spot was randomly flashed on a 5x5 grid for 0.6 s followed by 0.9 s pause (1.5 s inter-stimulus interval).

### Spike detection and sorting

Most analysis on recorded traces was performed with custom written routines by employing Igor Pro software (Wavemetrics, Lake Oswego, Oregon, USA). Matlab (Mathworks, Natick, MA, USA) was used for principal component analysis (PCA) while data clustering analysis was performed with a publically available KlustaKwik software (http://klustakwik.sourceforge.net).

Single units were first detected as threshold-crossing events while the threshold was set from 3 to 4.5 standard deviations from the baseline. The standard deviation was calculated according to Quiroga et al [33]. For tetrode recordings a lower by 30% threshold was used when synchronous events occurred on 2 or more traces. Care was taken that no artifacts were present in the trace. For each detected event 1.1 ms of each channel data was collected, 44 data points in total, 14 before and 30 after the negative peak.

For spike sorting first each channel data were reduced by principal component analysis (PCA) by employing Matlab software. Then spike classes by employing KlustaKwik software (http://klustakwik.sourceforge.net) for clustering of the first 2–5 principal components. If the results were unsatisfactory according to the absence of sufficient refractory period of ≥1.5 ms or poor cluster separation [34], additional feature based clustering was performed. The quality of sorting was verified with auto- and cross-correlograms [34].

### Receptive field area measurement

RF area was measured as a visual field area in which stimulus presentation induced a significant increase in the unit firing rate. An increase in the firing rate was measured either after the start of stimulus presentation (ON responses) or after the end of stimulus presentation (OFF responses). The significance was estimated at p < 0.01 level by employing a bootstrap method. We used a circular block version of *the bootstrap method* [35, 36] by following a procedure described here. First, a sample of background APs was obtained by taking all APs occurring either 0.4 s before stimulus in 1.5 s interval stimulation or 3–5 s before stimulus in 6–10 s interval stimulation. Then these short samples of 0.4 s or 3–5 s, obtained from one set of tests, were concatenated into a single series. Since the RF area was determined from at least 5 runs on either a 11x18-grid for 0.4 s samples or a 5x5-grid for 3–5 s samples, the obtained series of background APs was at least 375 s long (0.4 s * 11 * 18 * 5 or 3 s * 25 * 5). Next, AP numbers were counted in 100 ms bins. Finally, at least 200,000 samples of 5 consecutive bins, starting at randomly selected time points, were obtained for calculating the distribution of sample mean and for estimating the confidence interval of 99%. Since the original background AP sample is fairly large, ≥3750 bins of 100 ms, and auto-correlograms showed no correlation in the background AP sequence, no correction for bias was made [35, 37]. The background rate was estimated by counting the average rate of these APs before the stimulus presentation.

All results are presented as average ± SEM, for data statistical comparisons a non-parametric Kruskal-Wallis test was used.

## Results

Data presented here were collected from 16 rats. In all but two rat only a single recording site was used for analysis. Assuming that the appearance of the first visual responses indicate SC border (see Methods for details), all recorded neurons were located between 60 and 300 microns below SC surface, typically ~150 μm deep. In 3 cases these estimates were confirmed by lesions. In total, 24 units that met our selection criteria (see Methods for details) were analyzed, up to 3 units from a single recording site. Most units had very low spontaneous activity, <0.1 Hz.

**Fig 1:**
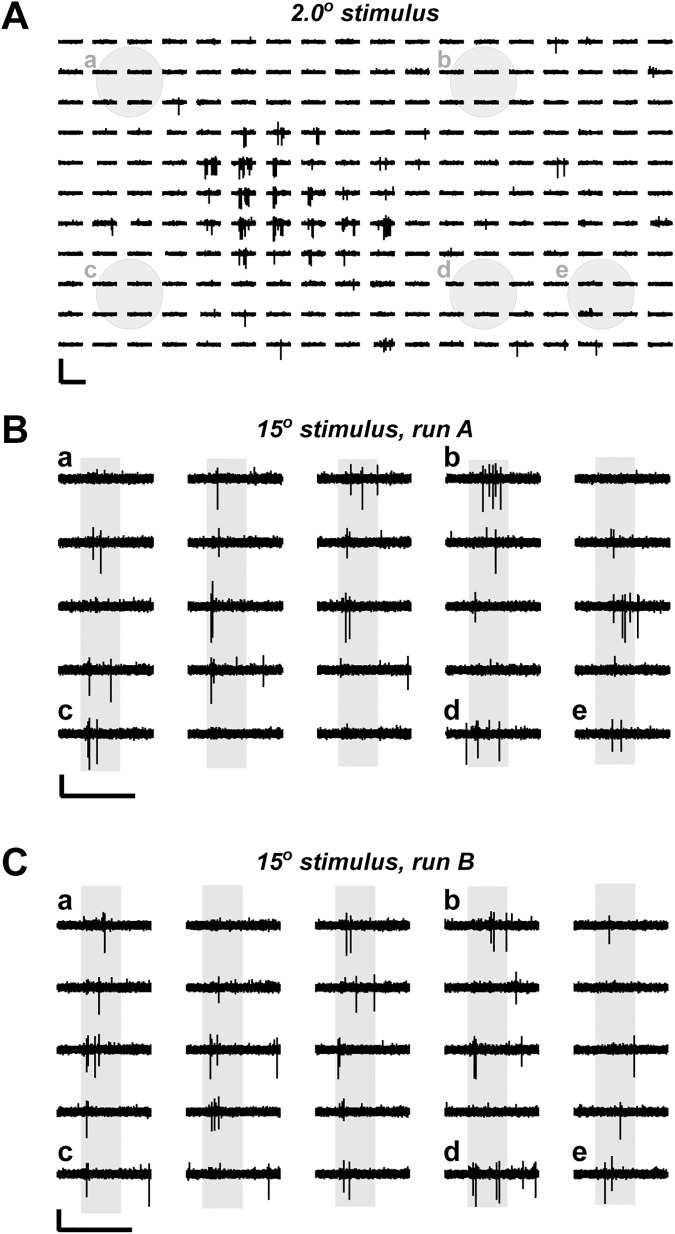
Large flashing spots evoked neuronal activity over much wider visual field area than small spots. *(A*) Trace samples of responses evoked by 2-degree spots. Trace position corresponds to the stimulus location on the monitor. Each trace is 2.0 s long; a 0.6 s stimulus was presented 0.4 s from the trace start. Grey filled circles with letters correspond to marked 15-degree stimulus locations shown in *B* and *C*. (*B)* and (*C*) Trace samples of responses evoked by 15-degree spots. Grey background bars indicate when the stimulus was presented. Letters by traces indicate responses corresponding to stimuli presented at locations shown in *A*. Scale bars are 250 μV and 2 s.

The initial observation that prompted to perform experiments described in this paper, was that in the neurons of the superficial SC layers the size of the excitatory RF apparently increased when larger visual stimuli were presented. An example of a set of traces obtained during such an experiment is shown in Fig 1.

**Fig 2:**
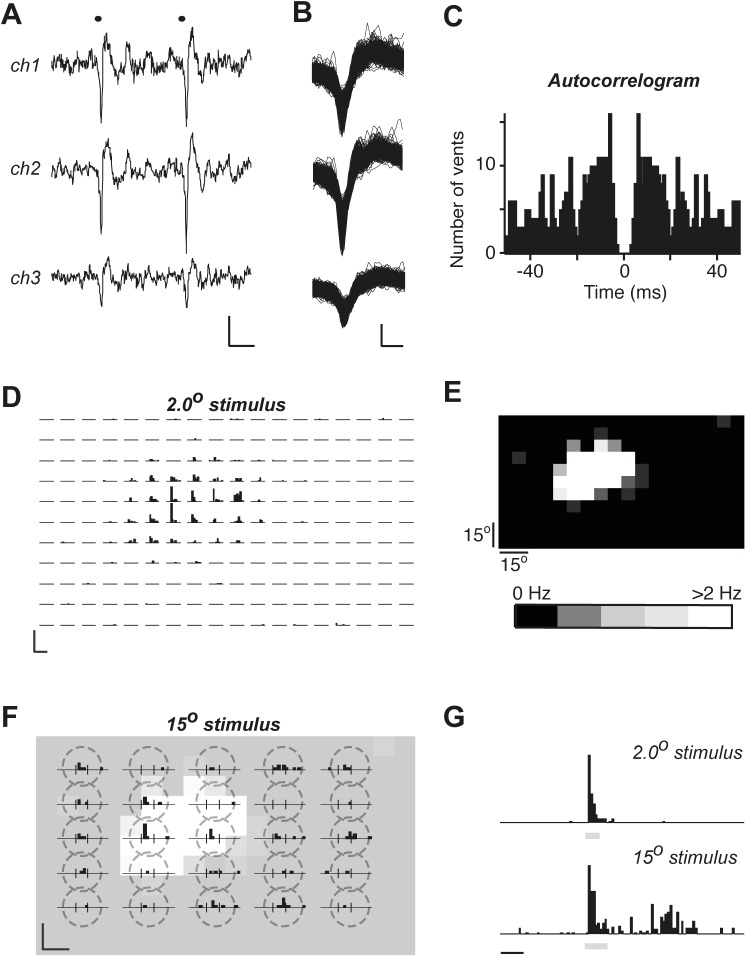
The apparent area of RF of a single unit increases with stimulus size. *(A*) A sample of two action potentials of a single unit recorded on 3 tetrode channels. Scale bars are 50 μV and 2 ms. (*B*) An overlap of all action potentials of a single unit shown for 3 tetrode channels. Scale bars are 50 μV and 0.25 ms. (*C*) An autocorrelogram for the single unit shown in *A*, and *B*, bar width is 1 ms. (*D*) Peristimulus time histograms (PSTHs) for this single unit for 2.0° stimuli. Each location was tested 6 times, bin width is 200 ms. Scale bars are 25 Hz and 2 s. (*E*) An average ON response firing frequency of the unit represented in grey scale to the location of the stimulus. The response was measured from 0 to 0.6 s from the stimulus start. The baseline frequency of 0.1 Hz was subtracted. Each square size is approximately 8° by 8°. (*F*) PSTHs for 15.0° stimuli. Each location was tested 2 times, bin width is 200 ms. Vertical thin black lines indicate the start and the end of the stimulus. Scale bars are 25 Hz and 2 s. The background image represents the ON response magnitude distribution taken from E and adjusted to match the location of 15° stimuli, indicated by broken line circles enclosing corresponding PSTHs. (G) Averaged responses to 2.0° and 15° stimuli demonstrate the presence of a slow component in the responses to 15° but not 2.0° stimuli. The response amplitudes were normalized to the peak (24 Hz in the 2.0° response and 5 Hz in the 15.0° response), bin width is 100 ms and the scale bar corresponds to 1 s. Grey bars below histograms indicate stimulus presentation.

In this experiment, first, a small, round, 2.0° diameter stimulus was used to determine the ‘classical’ excitatory RF (Fig 1A). As expected, stimulus-induced action potentials could be detected almost exclusively when the stimulus was flashed in a small area of the rat’s visual field, approximately 20° across. A different picture emerged when a much larger, 15° stimulus was used (Fig 1A and 1B). In this case, action potentials during stimulus presentation could be detected for almost all locations tested, covering most of the computer monitor, or >70° across. Grey filled circles in Fig 1A facilitate direct comparison between large and small stimulus locations. These circles are drawn to the size of 15° stimulus and demonstrate that large stimuli could evoke responses even in the areas where small stimuli could not induce any spikes in any location overlapping with the large stimulus. Although in most recordings several neurons contributed to overall activity, in this particular case almost all large action potentials could be attributed to a single unit/neuron. As shown in Fig 2A and 2B, these action potentials were stereotypical in shape and a clear refractory period could be identified in the auto-correlogram (Fig 2C).

A direct overlap of average spike counts in response to small stimulus presentations shows that large stimuli evoked response from areas, in which not a single action potential could be evoked in 6 runs (Fig 2D–2F). There was also a clear difference in the time course of responses to large and small stimuli: a substantial slow component was present in the response to large stimulus (Fig 2G). Such a slow component was present in the majority of responses to large stimuli.

**Fig 3:**
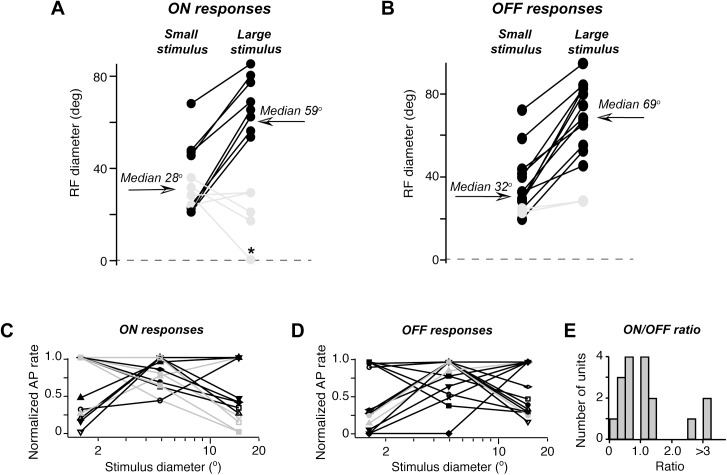
Summary of single unit properties that were tested for RF area increase with larger stimulus. *(A*) A plot of all tested single unit RF area of ON responses for small, 1.5° – 3.7° in diameter stimuli (filled circles on the left) and for 15° stimuli (filled circles on the right). RF area is represented as an average RF diameter (a square root of the RF area multiplied by a factor of 4/π). Median values of diameter are shown for both stimulus sizes. Units, for which no significant increase in RF area was detected, are in grey (only an increase in RF area that could not be accounted by stimuli overlap was considered as significant). An asterisk marks a unit, for which no significant responses to large stimuli could be detected. *(B*) The same as in A but for OFF responses. (*C)* and (*D*) The ON (*C*) and the OFF (*D*) response magnitude of all tested units plotted against the size of a round stimulus presented in the center of RF. Response magnitudes are normalized to the maximum response. Grey lines indicate units with no RF area increase while black lines correspond to the units with RF area increase (for ON responses in C and OFF responses in *D*). (E) The distribution of the ON to OFF response magnitude ratio in this figure neurons.

Such an increase in the visual field area, from which larger stimuli could evoke detectable responses was typical and could be reproduced in another 9 experiments, for total of 14 single units identified in these experiments. Although this phenomenon could be observed for both ON and OFF responses, that is, for action potentials induced by the stimulus onset (ON responses) and after the stimulus has ended (OFF responses), an RF area increase was more reliably reproduced for OFF responses (12 units of total 14 units) than for ON responses (8 units of total 13 units with ON responses, one unit did not have a detectable ON response). In 8 units, in which there was an increase in ON response area, the ‘classical’ RF area for ON responses was 1288 ± 410 degree^2^ (n = 8) while the ON response area to 15° stimulus was 3780 ± 443 degree^2^. For OFF responses an increase was from 1321 ± 317 to 4153 ± 462 degree^2^ (n = 12). The whole monitor area was 5625 degree^2^ and in the majority of cases it limited the area that could evoke responses to 15° stimulus; thus it is plausible that for large stimuli the actual responsive area was even larger. Although the shape of RF area was often irregular, to facilitate comparison with other papers [38, 39], these data can be expressed as a change in an average RF diameter (Fig 3A and 3B). The median RF diameter of ON responses increased from 28 degrees to 59 degrees while for OFF responses it increased from 32 degrees to 69 degrees. The median RF diameter stated here is somehow larger than it has been reported for adult rats (13 degrees in [38]) and for hamsters (~20 degrees in [39]), probably due to differences in the procedure used to measure RF area and relatively young age of animals used here (1.5–2.0 months or 45–60 days).

In spite of the increase in responsive area, the average spike rate was often reduced for large stimuli for both in RF and outside RF responses. Similarly to other species, most rat superficial SC neurons possess surround inhibition area that is attested by a decrease in the spike rates for large stimuli, which presumably exceed the excitatory RF area and the inhibitory surround reduces the in RF responses [20]. In line with these observations, during ON responses spike rates often first increased and then diminished when stimuli of increasing diameter were presented in the RF center (Fig 3A).

In our sample of 8 units, which possessed an increased ON response RF area for a 15° stimulus, in 5 units there was a decrease in the ON response magnitude when stimulus in RF size increased from 5° to 15° (Fig 3C). However, in 5 units that did not show an increase in the ON response RF area such a decrease in the ON response magnitude was observed in 4 out of 5 units. Although during OFF responses spike rates often increased even for relatively large stimuli (Fig 3D), in 5 units, for which the magnitude of OFF responses decreased when stimulus size increased from 5° to 15°, there was an increase in the OFF response area. In the remaining 7 units, possessing an increased OFF response area for large stimuli, there was an increase or no change in the OFF response magnitude when stimulus size increased from 5° to 15^**o**^. In addition, for the same stimulus size, the response magnitude outside RF was always reduced even for OFF responses (by 46%, n = 5). In our sample of 17 units, for which these tests were performed, there was no prevalence for either ON or OFF responses (Fig 3E). A complete list of RF area increases for each unit, including the main properties of these units, is provided in Table A and Table B in S1 Table.

There are several possible explanations for an apparent increase in the RF area for large stimuli. First, larger stimuli may overlap in part with the excitable RF and this overlap can be sufficient to induce a detectable response. Second, in fringe RF areas small stimuli may evoke only sub-threshold, i.e. undetectable responses, but, because of summation of synaptic inputs from much larger retinal area, large stimuli may be able to induce supra-threshold, i.e. detectable responses. Finally, due to light dispersion or reflections large stimuli can induce sufficiently strong changes in illumination in RF that will produce a response. Experiments described below aimed to address these possible causes.

Although the definition of RF may seem to imply clear RF borders, often the magnitude of neuronal responses decrease gradually when the distance from the stimulus to the RF center increases [40] with no clear-cut RF border. Although data shown in previous figures indicate no overlap between the ‘classical’ RF and the stimulus area, it is plausible that the number of scans (5–6) was insufficient to detect the fringe areas of RF that respond with low frequency firing. Two methods were used to better determine these fringe responses.

First, a two-dimensional Gaussian function was used to fit the average spike rate in response to stimulus onset and then the fit model was used to predict data for stimuli of different sizes presented in and outside RF [17, 41]. Only an excitatory part of the RF was used for the fit, thus presumably this model overestimated the spike rates during response to large stimuli and to stimuli outside RF because no inhibitory component is present in the model that would take into account the effects of inhibitory surround.

**Fig 4:**
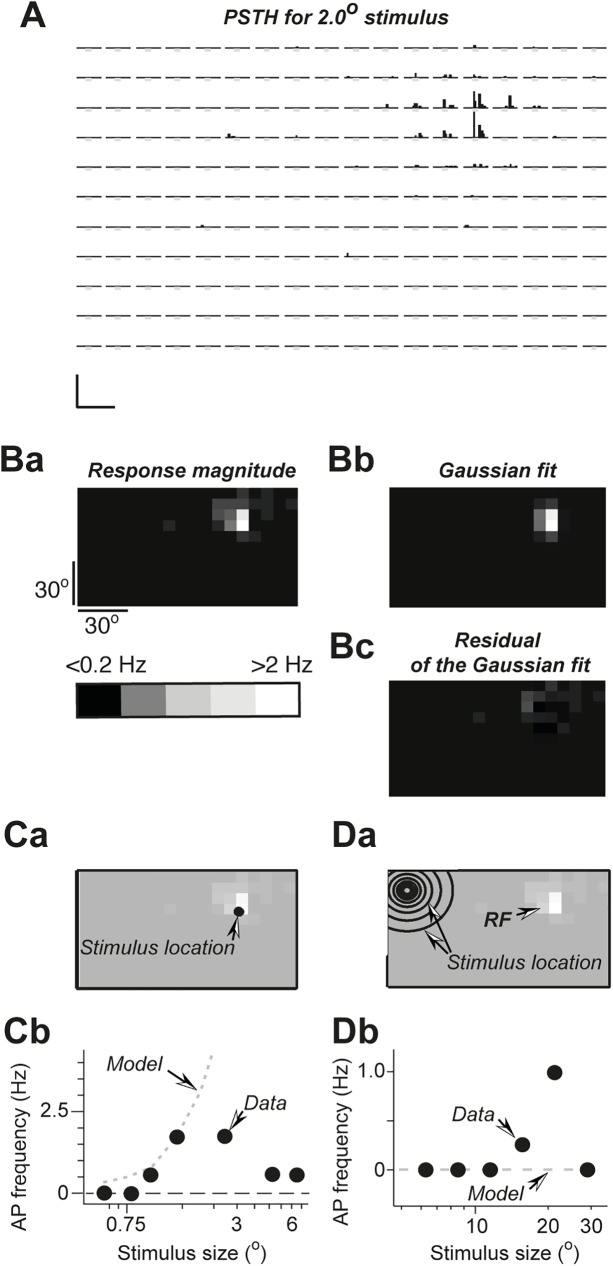
Gaussian fit model reproduces in part the responses in the RF but not outside RF. (*A*) PSTH for 2° spots. (*B*) The ON response magnitude (*Ba*), its Gaussian fit (*Bb*) and the residual of the fit (*Bc*). The response magnitude for all panels is represented in grey scale shown in the lower left corner. (*C*) Responses to the spots of different sizes flashed in RF. Spot location and its size are shown as black circles (*Ca*). The real response data (filled circles) and the model predictions (broken line) are plotted as response magnitude against the spot size (*Cb*). (*D*) The same as in *C* for the outside RF stimulus.

Fig 4A shows and example of peri-stimulus time histogram (PSTH) of responses of a single unit to 2° stimulus. A Gaussian fit to the peak firing rate of the ON responses was calculated by averaging spike rate between 0.1 s and 0.3 s from the stimulus start (Fig 4Ba). The fit reproduced relatively well the distribution of the peak firing rates (Fig 4Bb) and real data deviation from the fit was less than 20% of the peak value in the RF center (Fig 4Bc). Next, the obtained fit function was used to predict the dependence of spike rates in response to stimuli of different sizes presented either in the RF or outside RF. In this model it was assumed that the number of evoked action potentials is proportional to the product of the stimulus area and the action potential rate predicted by the fit. For small stimuli of less than 3° in diameter this fit model reproduced quite well experimental data (Fig 4Cb). Since our Gaussian fit model did not include a component for the inhibitory surround, the model heavily over-estimated the reduced firing rates above 3°. In contrast, for stimuli outside RF our model predicted virtually no action potentials even for the largest stimuli in spite of the absence of inhibitory component in the model (Fig 4D). Similar results were obtained for all units tested with this method (n = 5).

**Fig 5:**
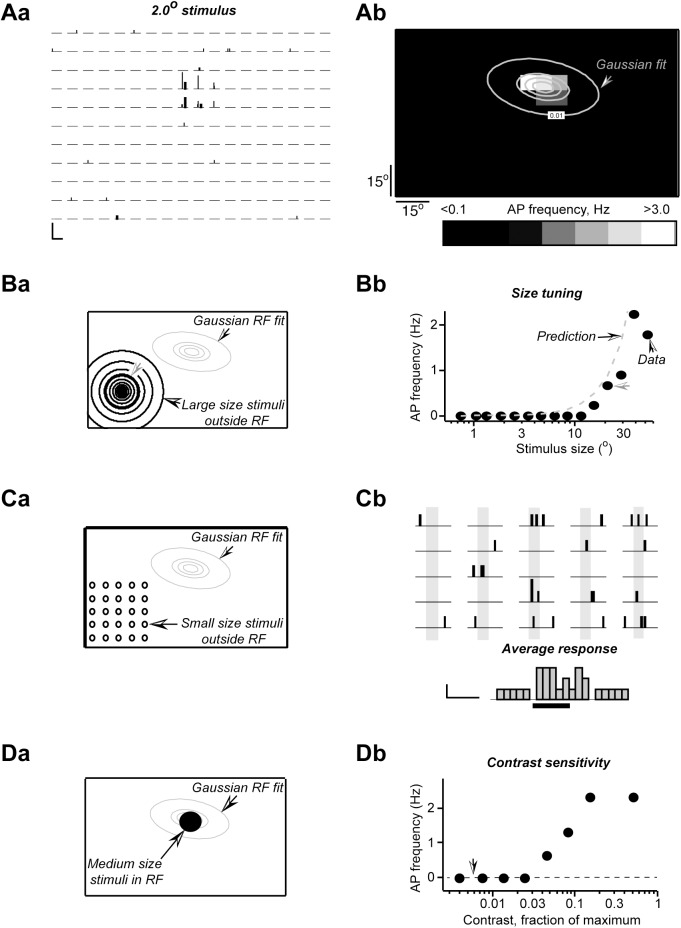
An increase in spike rate during small stimulus presentation but not reflections or light diffusion can predict responses to large stimuli outside RF. *(A*) PSTH for 2° (*Aa*) and the ON response magnitude (*Ab*), represented in grey scale with overlaid its Gaussian fit shown as grey lines. The outside line corresponds to the Gaussian fit prediction of 0.01 Hz response magnitude. (*B*) The ON response magnitude to the outside RF stimuli, the location of which is shown in *Ba* as black circles, plotted against the stimulus size (*Bb*). A thicker circle indicates the stimulus size that is marked in the plot in *Bb* by a grey arrow. The prediction response magnitude (a broken grey line in Bb) was obtained by extrapolating data of experiment shown in *C*. For details see the main text. (*C*) The location of 2° stimuli and the corresponding PSTH. Each spot was tested 50 times, 1250 tests in total. The average of all 1250 tests is shown below PSTH. Light grey bars in PSTH indicate when stimulus was on. Scale bars are 0.01 Hz and 0.5 s. (*D*) The location of 15.0° stimuli of different contrast (*Da*) and the magnitude of obtained responses plotted against stimulus contrast (*Db*). An arrow in the plot in *Db* indicates the maximum expected change in illumination due to light diffusion and reflections.

It is clear that, for ON and OFF responses, a two-dimensional Gaussian fit is only an approximate model of the spike rate dependence on the visual stimulus location and a more direct estimate of these spike rates beyond the central part of the RF is desirable. To this end, in 7 recording sessions, in addition to RF determination with a small, 2–4 degree visual stimuli, repeated on a grid for 5 to 9 times as shown in examples of Figs 2 and 4, a small section of the outside RF area was scanned >20 times with the same size small stimulus in order to reveal these low frequency responses (Fig 5).

In this series of experiments, first the RF was determined with a usual 11 by 18 grid; each location of the grid being tested at least 5 times (PSTH in Fig 5A). Due to a large number of tests required, it was not feasible to test each location of such a large grid >20 times, that would resulted into >3500 visual stimulus presentations (11X18X20). Therefore, the outside RF area, where large outside RF stimuli were presented and responses to such stimuli were recorded (Fig 5B), was scanned 50 times to detect rare events. The results of such an experiment are shown in Fig 5B and 5C. Fig 5B shows that for this particular unit there were detectable responses to large, over 10° spots, flashed for 1 s well outside the Gaussian fit area of 0.01 Hz; that is the area in which 2° stimulus would evoke action potentials at an average rate of 0.01 Hz. Assuming that the rate of action potentials is proportional to the stimulus area, such a fit predicts an average rate of less than 1 Hz to stimuli of 20° or smaller. However, as indicated by a grey arrow in Fig 5Bb, the actual response was close to 2 Hz for a 20° stimulus. Although repeated presentation of 2° stimulus on the outside RF grid (Fig 5Ca) induced very few action potentials that seemed to occur randomly (Fig 5Cb), the average, which is shown at the bottom of Fig 5Cb and corresponds to 50 x 25 = 1250 stimulus presentations, indicated though small but a clear response. For this integrated average over the whole section of the visual field, non-parametrical statistical tests showed a statistically significant increase in the firing frequency from 0.008 Hz to 0.016 Hz (p < 0.06). We assumed that we could average these responses corresponding to different stimuli locations; more specifically, we assumed that responses to small stimuli presented in the selected visual field area were relatively homogenous and the average reflected the properties of a response to a visual stimulus located anywhere inside this section of the visual field. This assumption is supported by the fact that such a tiny increase in the firing rate is sufficient to explain the observed responses as shown by a plot that compares the prediction (a grey broken line in Fig 5Bb) and the experimental data (black filled circles in Fig 5Bb). The prediction was calculated assuming that the response magnitude, i.e. the action potential frequency, is proportional to the stimulus area and the surround inhibition was not taken into account. The graph shows that predicted rates are higher than the actual data points. This is not surprising since inhibition was not accounted for in the prediction. In 5 out of 7 units such tests yielded prediction rates that could account for at least 50% of the observed response rates. It is clear that we are dealing with very low rates and even such large number of tests is insufficient to obtain very reliable results but it provides evidence that even these small increases in the action potential firing rates can predict quite well the magnitude of responses to large stimuli.

Finally, to exclude that small changes in illumination in RF caused by a distant large stimulus may account for the observed outside RF responses, the following tests were performed. First, changes in illumination caused by a 20° circle were estimated for a location on the monitor that was away from the stimulus by approximately one half of the monitor height or ~37°. The local changes in illumination were <1/160^th^ of the illuminance at the stimulus location (16–32 lx versus 0.1 lx), or <1% of the stimulus contrast used here for the tests. Thus, if the rat’s eye responds to these tiny changes in illumination, we should be able to directly detect these responses as an increase in the spike rate. To this end, contrast sensitivity was measured for stimuli that covered most of the ‘classical’ RF (Fig 5D). Since reflections or light dispersion cannot create an optimal stimulus, a stimulus that roughly covers the center of RF should imitate well the changes in illumination caused by an outside RF stimulus. Typical results of such tests are shown in Fig 5D. Although this unit was quite sensitive and responded well to a stimulus, the contrast of which was only 5% of the maximum, such sensitivity was far too low to account for the observed responses (the required sensitivity is shown by an arrow in the plot of Fig 5Db). In 5 other tested neurons the contrast sensitivity threshold varied from 2.5 to 15% of the maximal obtainable contrast. Thus, it can be concluded, that typically the sensitivity of neurons to small changes in illumination was insufficient to account for the observed outside RF responses.

## Discussion and conclusions

We demonstrate that large stationary stimuli outside the classical RF can evoke detectable responses in the SC superficial layer neurons of urethane-anaesthetized rats. These responses could not be explained neither by partial overlap of the stimulus with the excitatory RF nor by reflections or light dispersion from large stimuli. These data indicate that the SC superficial layers neurons are capable of visual stimuli integration well beyond the borders of their classical RFs.

Extra-classical RF responses are well described in the primary visual cortex and lateral geniculate nucleus of monkeys and some other species [6, 9]. Responses to visual stimuli presented in RF can be both suppressed and enhanced by stimuli outside RF, the direction of modulation depends on the outside stimulus position and the alignment with the RF stimulus. Furthermore, even in the absence of the RF stimulus, the correlated activity of the spontaneous firing may change [12]. These phenomena are thought to reflect the need for visual system to integrate visual images over large visual field areas in order to recognize complex images.

Although such evidence is scarce for SC, it has been reported that responses in neurons of the uppermost layer of SC are modulated by changes in illumination of the surround background, indicating that these neurons are also capable of integrating extra-classical RF stimuli [23]. However, neurons recorded for this study were located at least 50 microns below SC surface, thus they are located below the uppermost SC layer. The properties of RF of these deeper neurons have been extensively studied since early days of vision neuroscience [16, 19, 22, 42]. At least some early investigators did test large stimuli outside RF and it was reported that no responses could be evoked [19], no other study suggested of the presence of responses to stimuli outside RF. There are several possible explanations why such type of responses has not been reported so far. First, the frequency of evoked action potentials of these responses is typically very low, several fold lower than the responses induced by small size stimuli in RF. This reduction in the action potential frequency is caused by two factors. First, large stimuli typically evoke lower magnitude responses (Fig 3). Second, even for the same size of stimuli the response magnitude outside RF was only about half of the magnitude in RF. Therefore, it is easy to dismiss such weak responses as an aberration or an event of spontaneous activity. Finally, many investigators used moving stimuli that may not produce this type of response.

A clue about the nature of outside RF responses is given by limited correlation between the magnitudes of responses to small and large stimuli outside RF (Fig 5). These experiments show that outside RF a very weak responses may exist that may become significant when stimulus size increases. Of course, this hypothesis assumes that the response magnitude is proportional to the stimulus area and there is some evidence for that (Fig 4Cb) even though most SC neurons seem to be non-linear [17]. This hypothesis also assumes that SC neurons receive sparse, probably indirect inputs from distant areas of retina that may be responsible for such outside RF responses. Such inputs would enable visual stimuli integration over large visual field areas.

Indeed, there is evidence that SC neurons are able to rapidly integrate visual stimuli over very wide visual field areas. For instance, in cats at least some SC neurons are sensitive to collision stimulus [43]. Such collision-sensitive neurons can differentiate if a rapidly expanding stimulus is representing or not a danger as an object approaching on a collision course; in addition these neurons can predict time to collision [44]. All these operations require very rapid integration of images from very wide areas of the visual field. Although no such neurons have been reported yet for rodents [18], these data suggest that many SC neurons may be capable of wide range visual stimuli integration. Data presented here confirm that these neurons are capable of visual stimuli integration from areas >50 degrees apart.

It is plausible, that urethane anesthesia used in our experiments changed the balance between inhibition and excitation [45] and normally sub-threshold responses became supra-threshold. Some researchers have suggested that urethane leaves synaptic transmission largely intact [46] but the effects of urethane may depend on the brain area [45, 47]. It has been reported that in rats urethane alters EEG signals evoked by visual stimuli [48] although other anesthetics also affect such visual responses [49, 50]. To the best of our knowledge, there is no direct comparison of RF properties in SC in awake and anesthetized rodents. However, it has been shown that in mice urethane anesthesia reduced the magnitude of responses to looming stimuli and eliminated cortical effects [18]. On the other hand, in LGN and cortical neurons the basic RF properties are not changed by anesthesia [51].

One may speculate that, because of urethane anaesthesia, the reported here responses in SC neurons to visual stimuli outside RF are somehow related to much larger RF area that is observed in young animals [38, 39]. It is plausible that in adult rats these distant connections, clearly functional in young but not adult animals, become more active under urethane anesthesia but under normal circumstances they do not serve any role except for emergency situations like glaucoma or scotoma, when RF area is increased [52, 53]. Nevertheless, the results presented here demonstrate that in the superior colliculus superficial layers there are functional connections from very distant to RF areas that can generate action potentials under certain conditions and may serve as a basis for integrating visual stimuli over very wide areas of the visual field.

We conclude that the superior colliculus neurons receive inputs from very large retinal areas. It is plausible that all inputs from extra-classical RF area are indirect. However, under certain circumstance they can evoke action potentials and can help to integrate visual stimuli over very large visual field areas.

## Supporting information

S1 Table RF size changes for single units and the main properties of these units. Two tables, table A, spontaneous firing and ON responses, and table B, OFF responses, include all units for which the receptive field area for two different stimulus sizes was measured. For small stimuli grid step was 7.5°, thus all area numbers are multiples of a grid unit area of 56.25 degrees^2^. Large stimuli grid step was 15°, thus area numbers are multiples of 225°. RF area included only grid elements, in which the average AP rate was significantly higher than the background AP rate, p < 0.01, confidence intervals for the background AP rate were estimated with a bootstrap method as described in the Methods. For large stimuli, an increase in the RF area was considered significant only if it could not be explained by the stimulus overlap with the small stimulus grid elements, in which small stimulus evoked a significant response. ON and OFF time interval indicates time interval after the stimulus start (for ON responses) or the stimulus end (for OFF responses) during which the average AP rate during response was measured. These interval were selected to represent the most significant part of the response and, for a single unit, were the same for all stimulus sizes. Since small stimulus size was selected to have unit sensitivity close to optimal, the small stimulus size used to measure RF area in response to small stimulus differed for different units and it is shown in the second column of the tables.
(DOCX)
