# Supplementary material for: Rat superior colliculus neurons respond to large visual stimuli flashed outside the classical receptive field
Source: PLoS One. 2017 Apr 5;12(4):e0174409. doi: 10.1371/journal.pone.0174409 (PMC5381878; doi:10.1371/journal.pone.0174409)
Supplement: S1 Table — Two tables, table A, spontaneous firing and ON responses, and table B, OFF responses, include all units for which the receptive field area for two different stimulus sizes was measured. For small stimuli grid step was 7.5°, thus all area numbers are multiples of a grid unit area of 56.25 degrees2. Large stimuli grid step was 15°, thus area numbers are multiples of 225°. RF area included only grid elements, in which the average AP rate was significantly higher than the background AP rate, p < 0.01, confidence intervals for the background AP rate were estimated with a bootstrap method as described in the Methods. For large stimuli, an increase in the RF area was considered significant only if it could not be explained by the stimulus overlap with the small stimulus grid elements, in which small stimulus evoked a significant response. ON and OFF time interval indicates time interval after the stimulus start (for ON responses) or the stimulus end (for OFF responses) during which the average AP rate during response was measured. These interval were selected to represent the most significant part of the response and, for a single unit, were the same for all stimulus sizes. Since small stimulus size was selected to have unit sensitivity close to optimal, the small stimulus size used to measure RF area in response to small stimulus differed for different units and it is shown in the second column of the tables. (DOCX) [file pone.0174409.s001.docx]

**S1 Tables. RF size changes for single units and the main properties of these units.**

Two tables, table A, spontaneous firing and ON responses, and table B, OFF responses, include all units for which the receptive field area for two different stimulus sizes was measured. For small stimuli grid step was 7.5^o^, thus all area numbers are multiples of a grid unit area of 56.25 degrees^2^. Large stimuli grid step was 15^o^, thus area numbers are multiples of 225^o^. RF area included only grid elements, in which the average AP rate was significantly higher than the background AP rate, p < 0.01, confidence intervals for the background AP rate were estimated with a bootstrap method as described in the Methods. For large stimuli, an increase in the RF area was considered significant only if it could not be explained by the stimulus overlap with the small stimulus grid elements, in which small stimulus evoked a significant response. ON and OFF time interval indicates time interval after the stimulus start (for ON responses) or the stimulus end (for OFF responses) during which the average AP rate during response was measured. These interval were selected to represent the most significant part of the response and, for a single unit, were the same for all stimulus sizes. Since small stimulus size was selected to have unit sensitivity close to optimal, the small stimulus size used to measure RF area in response to small stimulus differed for different units and it is shown in the second column of the tables.

Table A. Spontaneous firing and ON responses

| Unit (date and name) | Spontaneous firing frequency (Hz) | Small stimulus diameter | ON time interval | 2.1^o^ – 3.7^o^ stimulus ON RF area, degrees^2^ | 15^o^ stimulus ON RF area, degrees^2^ | 1.5^o^ ON Hz | 3.7^o^ ON Hz | 15^o^ ON Hz |
| --- | --- | --- | --- | --- | --- | --- | --- | --- |
| **2014.12.26A cl7** | 0.1-0.2 | 1.5^o^ | 0-0.5 s | **618.75** | **0** | 4.7 | 2.0 | 0.0 |
| **2014.12.26A cl5_3t1** | 0.16 | 1.5^o^ | 0-0.5 s | **787.5** | **225** | 1.9 | 1.5 | 0.4 |
| **2014.12.29A cl18_cl1** | 0.045 | 3.7^o^ | 0.-0.3 s | **450** | **2475** | 3.0 | 2.0 | 1.2 |
| **2014.12.29A DT3Ch3Fx re-sorted** | 0.025 | 3.7^o^ | 0.1-0.3 s | **337.5** | **2250** | 1.2 | 1.7 | 4.0 |
| **2015.02.04A**  **Unit1, cl3+cl17** | 0.08 | 3.7^o^ | 0.-0.2 s | **393.75** | **3375** | 2.0 | 11 | 2.7 |
| **2015.06.13**  **Unit1, DT3_cl3cl5** | 0.03 – 0.18 | 3.7^o^ | 0.1-0.3 s | **450** | **675**  *(overlap with RF 3.7^o^)* | 1.1/0.5 | 1.5 | 3.0/1.5 |
| **2016.07.07 unit1A** | 0 | 3.7^o^ | 0.1-0.4 s | **56.25** | **0** | 0 | 0  0.4 in RF center | 0 |
| **2016.07.07 unit2A** | 0.035 – 0.23 | 3.7^o^ | 0.1-0.4 s | **1743.8** | **3712.5** | 1.6 | 3.3 | 3.0/4.2 |
| **2016.07.07 unit34A** | 0.033 | 3.7^o^ | 0.1-0.4 s | **337.5** | **3037.5** | 0.0 | 0.8 | 0.8 |
| **2016.07.28** | 0.085 – 0.35 | 2.0^o^ | 0.1-0.4 s | **3656.3** | **5737.5** | 0.85 | 6.0 | 2.7 |
| **2016.08.03d240** | 0.035 – 0.07 | 2.0^o^ | 0.1-0.6 s | **1800** | **47.25** | 11.5 | 7.0 | 3.7 |
| **2016.09.27 cl7cl10** | 0 - 0.02 | 3.7^o^ | 0.1- 0.5 s | **618.75** | **675** | 0.4 | 0.4 | 0-0.1 |
| **2016.10.18, cl2** | 0.15 | 2.0^o^ | 0.1- 0.3 s | **1631.3** | **5062.5** | 5 | 4.2 | 2.0 |
| **2016.10.25, DT2.2 cl5t1** | 0.1 – 0.01 | 2.0^o^ | 0.1- 0.3 s | **1012.5** | **337.5** | 1.6 | 1.0 | 0 |

Table B. OFF responses

| Unit (date and name) | OFF time interval | Small stimulus diameter | 2.1^o^ – 3.7^o^ stimulus OFF RF area, degrees^2^ | 15^o^ stimulus OFF RF area, degrees^2^ | 1.5^o^ OFF Hz | 3.7^o^ OFF Hz | 15^o^ OFF Hz |
| --- | --- | --- | --- | --- | --- | --- | --- |
| **2014.12.26A cl7** | 0-1.0s | 1.5^o^ | ***900*** | ***3600*** | 6.5 | 7.0 | 2.1 |
| **2014.12.26A cl5_3t1** | 0-1.0s | 1.5^o^ | ***675*** | ***5400*** | 4.4 | 4.5 | 1.8 |
| **2014.12.29A cl18_cl1** | 0.-0.3s | 3.7^o^ | ***337.5*** | ***2250*** | 2.5 | 2.0 | 1.2 |
| **2014.12.29A DT3Ch3Fx re-sorted** | 0.1-0.2s | 3.7^o^ | ***506.25*** | ***2475*** | 8.5 | 3.3 | 2.5 |
| **2015.02.04A**  **Unit1, cl3+cl17** | 0.-0.2s | 3.7^o^ | ***450*** | ***675***  *(2700 for 0-1.0s)* | 2.5 | 8.0 | 2.7 |
| **2015.06.13**  **Unit1, DT3_cl3cl5** | 0.1-0.3s | 3.7^o^ | ***2756.3*** | ***5625*** | 1.0/0.8 | 5.0 | 6.5/5.0 |
| **2016.07.07 unit1A** | 0-0.5s | 3.7^o^ | ***675*** | ***4387.5*** | 0 | 1.0  *(2 in RF center)* | 0.25 |
| **2016.07.07 unit2A** | 0-0.5s | 3.7^o^ | ***1293.8*** | ***5400*** | 0.2 | 2.0/2.7 | 2.5/5.2 |
| **2016.07.07 unit34A** | 0-0.5s | 3.7^o^ | ***731.25*** | ***5062.5*** | 0.2 | 0.72 | 0.47 |
| **2016.07.28** | 0-1.0s | 2.0^o^ | ***4162.5*** | ***7087.5*** | 0.5 | 1.2 | 1.5 |
| **2016.08.03d240** | 0.1-0.6s | 2.0^o^ | ***1406.3*** | ***3712.5*** | 0 | 0.7 | 0.7 |
| **2016.09.27 cl7cl10** | 0.1- 0.6s | 3.7^o^ | ***506.25*** | ***675*** | 0.1 | 0.4 | 0.1 |
| **2016.10.18, cl2** | 0.1- 0.3s | 2.0^o^ | ***1575*** | ***3375*** | 4.0 | 2.2 | 1.2 |
| **2016.10.25, DT2.2 cl5t1** | 0.1- 0.5s | 2.0^o^ | ***900*** | ***1687.5*** | 2.2 | 0.4 | 0 |
